# Excessive remifentanil during total intravenous anesthesia is associated with increased risk of pain after robotic thyroid surgery

**DOI:** 10.1371/journal.pone.0209078

**Published:** 2018-12-14

**Authors:** Hyung-Chul Lee, Ho-Geol Ryu, Hyung-Jun Kim, Yoonsang Park, Soo Bin Yoon, Seong Mi Yang, Hye-Won Oh, Chul-Woo Jung

**Affiliations:** Department of Anesthesiology and Pain Medicine, Seoul National University College of Medicine, Seoul National University Hospital, Seoul, Republic of Korea; Cleveland Clinic Lerner College of Medicine of Case Western Reserve University, UNITED STATES

## Abstract

The widespread use of remifentanil during total intravenous anesthesia (TIVA) has raised concerns about the risk of postoperative remifentanil-associated pain. Although a recent meta-analysis suggests that remifentanil-associated pain is unlikely to occur in patients with TIVA because of the protective effect of co-administered propofol, the evidence is not conclusive. We retrospectively assessed 635 patients who received robotic thyroid surgery under TIVA to evaluate the risk of remifentanil-associated pain. Postoperative pain was evaluated using 11-point numeric rating scale (NRS). Time dependent Cox proportional hazards regression analysis was used to determine the risk factors of treatment-requiring pain (NRS > 4) during the first 48 postoperative hours. Postoperative pain rapidly decreased, and treatment-requiring pain remained in 12.8% (81 out of 635) of patients at 48 hours postoperatively. After adjusting for the time-dependent analgesic consumption, intraoperative use of remifentanil > 0.2 mcg/kg/min was a positive predictor of postoperative pain with a hazard ratio of 1.296 (95% C.I., 1.014–1.656, *P* = 0.039) during 48 hours after surgery. In conclusion, excessive use of remifentanil during TIVA was associated with increased risk of pain after robotic thyroid surgery. Prospective trials are required to confirm these results and determine whether decreasing remifentanil consumption below the threshold can reduce postoperative pain.

## Introduction

Remifentanil is one of the most commonly used analgesic in recent anesthesia practice due to its rapid onset and offset. However, with the widespread use of remifentanil, there are concerns about new problems related to postoperative pain. Due to the short duration of action, the analgesic effect of remifentanil rapidly disappears with the termination of infusion, raising the issue of proper postoperative analgesia. In addition, the association between excessive remifentanil use during surgery and acute opioid tolerance (AOT) and/or opioid-associated hyperalgesia (OIH) after surgery has been suggested by many prospective studies and subsequent meta-analysis and systematic reviews [1–5].

Total intravenous anesthesia (TIVA) has become popular in recent years and usage of remifentanil has substantially increased more than when it was used as an adjuvant in inhalation anesthesia. Especially, the widespread use of bispectral index (BIS) monitor enabled titrated and reduced administration of propofol, which then increased the use of remifentanil to compensate for anesthetic synergy. Concerns about the risk of remifentanil-associated pain after TIVA are increasing, however studies on remifentanil-associated pain in TIVA are lacking [6–10]. Recent meta-analysis suggests that remifentanil-associated pain is unlikely to occur in patients with TIVA because of the protective effect of co-administered propofol, however the evidence is not robust due to the small sample size and inconsistent analytical methods of the enrolled studies [4].

We retrospectively evaluated patients who underwent robotic thyroid surgery to assess whether excessive remifentanil use in TIVA increases postoperative pain. The primary goal of this study is to confirm that the postoperative pain scores adjusted by analgesic use differ depending on the amount of remifentanil used during TIVA.

## Materials and methods

This retrospective study was approved by the Institutional Review Board of Seoul National University Hospital, Seoul, Korea (IRB number: H-1612-137-821) on 11 January 2017. Informed consent was waived due to the retrospective design of the study. This manuscript adheres to the *STROBE* guideline.

### Subjects

Adult patients who underwent robotic thyroid surgery between January 2011 and October 2016 were included in the study. Exclusion criteria were: 18 years of age or younger; obesity (body mass index > 30 kg/m^2^); drug or alcohol abuse history; renal or hepatic dysfunction; mental disorder; allergies or hypersensitivity to analgesics; current use of analgesics due to acute or chronic pain; lack of ability to respond to pain questions; incomplete medical record; and enrollment in another clinical trial during the study period.

### Anesthesia and surgery

Patients arrived at the operating room without premedication. Routine monitoring such as electrocardiography, noninvasive blood pressure, pulse oximetry and BIS monitor (BIS Vista^®^ Covidien, Dublin, Ireland) was applied to patients before anesthesia induction. Anesthesia was induced and maintained by effect-site target-controlled infusion (TCI) of propofol and remifentanil. After preoxygenation, target effect-site concentration (Ce) of propofol and remifentanil were set at 3–4 mcg/mL and 5–6 ng/mL, respectively. When the patient did not respond to verbal commands and BIS became less than 60, rocuronium 0.6–0.9 mg/kg was administered. After disappearance of twitch response to train of four stimulation, the trachea was intubated, and mechanical ventilation was initiated with an inspired oxygen fraction of 0.4–0.5 and a tidal volume of 6–8 mL/kg. Rocuronium 0.15 mg/kg was intermittently administered to maintain neuromuscular block during surgery under the ulnar nerve monitoring. When the recurrent laryngeal nerve was monitored intraoperatively, additional rocuronium was not administered and if required, reversal of neuromuscular block was performed with neostigmine 0.04 mg/kg and glycopyrrolate 0.01 mg/kg before start of tissue dissection. After completion of thyroidectomy, rocuronium was administered again until the end of surgery and reversal was performed with neostigmine 0.04 mg/kg and glycopyrrolate 0.01 mg/kg.

Robotic thyroid surgery was performed using the bilateral axillo-breast approach with the da Vinci Robot System (Intuitive Surgical, Inc., Mountain View, CA, USA) [11]. Incision was made on both axillary and periareolar regions. After insertion of ports to the incised skin, a skin flap was made from the ports to the neck. The flap was then extended to the thyroid cartilage, the clavicle, and the sternocleidomastoid muscle. After the robot was docked, dissection of the tissues and thyroidectomy were done with electrocautery and ultrasonic device.

After surgery, the patient was transferred to the postanesthesia care unit (PACU) without patient-controlled analgesia. The patient was transferred to the ward after observation at PACU for 30 minutes to 1 hour.

### Evaluation and intervention of pain

Pain was evaluated with an 11-point (0–10) numeric rating scale (NRS). Point 0 was painless state and point 10 was the worst pain imaginable. This pain scale was explained to the patient before the day of surgery and evaluated by nurses every 8 hours after surgery. According to our institution’s protocol, postoperative analgesic was administered when the NRS was greater than 4 and the patient accepted the offered pain medication. Therefore, pain with an NRS greater than 4 was defined as “treatment-requiring pain”.

Both opioid and non-opioid drugs were intravenously used for postoperative analgesia. Opioid analgesics included fentanyl and meperidine, which were mainly used at PACU. The dose was at the discretion of the on-duty anesthesiologist. Non-opioid drug was intravenous ketorolac 30 mg and was used in the wards according to the surgeon’s decision.

### Data acquisition

All data were retrieved from the electronic medical recording system of our institution. Patient age, weight, height, anesthesia duration and total consumption of propofol and remifentanil during surgery were recorded from the electronic anesthesia chart. Pain score, analgesic consumption and time of evaluation or intervention at PACU and ward were recorded from the PACU chart and ward record, respectively, until 48 hours after surgery.

### Sample size estimation

The incidence of postoperative pain was reported to be 15% (7 out of 47) between 24 and 48 hours after robotic thyroid surgery in a previous report [12]. We assumed that consumption of remifentanil, propofol and analgesic would be independently related with the presence of treatment-requiring pain within 48 hours after surgery. Sample size calculation was based on Cox proportional hazards regression analysis to detect at least 3 independent variables. According to the simplified formula by Peduzzi and colleagues [13], the minimum sample size was calculated as follows:

N = 10 x number of predictors / proportion of positive cases = 10 x 3 / 0.15 = 200.

### Statistical analysis

All data generated or analyzed during this study can be found in S1 File.

Continuous variables were expressed as median (interquartile range). Categorical variables were expressed as number (%). Descriptive statistic was used for patient characteristics.

Cox proportional hazards regression analysis was performed to evaluate the relationship between risk factors and the presence of treatment-requiring pain after surgery. The risk factors included sex, age, weight, duration of anesthesia, intraoperative remifentanil consumption, intraoperative propofol consumption and postoperative analgesic consumption.

Intraoperative remifentanil consumption was tested as continuous variables such as weight-adjusted dose (mcg/kg) or average infusion rate (mcg/kg/min). The values were also tested as category variables that were classified according to previously reported cutoffs: 0.1 mcg/kg/ min [5], 0.2 mcg/kg/min [2], 0.25 mcg/kg/min [2], and 50 mcg/kg [3]. Intraoperative propofol consumption was tested as continuous variables such as weight-adjusted dose (mg/kg) or average infusion rate (mg/kg/min).

Postoperative analgesic consumption was converted to intravenous morphine 10 mg equivalent dose (MED10). Doses of fentanyl and meperidine were converted using opioid-dose-conversion table: the potency of intravenous fentanyl 100 mcg or meperidine 75 mg was considered to be the same as intravenous morphine 10 mg [14]. Intravenous ketorolac 30 mg was considered equipotent to intravenous morphine 10 mg because of similar number-needed-to-treat (about 3) according to the Oxford league table of analgesic efficacy [15]. The amount of analgesic consumption measured as MED10 was summed for 48 postoperative hours and used as a continuous variable risk factor in the analysis.

The outcome variable was the presence of treatment-requiring pain within 48 hours after surgery. The first time when the NRS became 4 or less continuously was considered as the time of treatment-requiring pain disappearance. If the NRS did not reach 4 or less within 48 hours after surgery, the case was treated as censored.

Multivariable Cox proportional hazards regression analyses were performed with forward stepwise method using 1 of 6 remifentanil risk factors, 1 of 2 propofol risk factors, and analgesic consumption as independent variables and the presence of treatment-requiring pain as a dependent outcome. Propofol and remifentanil risk factors were paired with each other and tested 12 times in total. The assumption of proportional hazards was tested with log minus log survival plots and Schoenfeld residuals. If violation of model assumption was confirmed by Spearman correlation test between the time factor and Schoenfeld residuals of identified risk factors, time dependent Cox proportional hazards regression analysis was performed.

Finally, comparison of remifentanil consumption categories identified as significant in the regression analysis was performed using Mann-Whitney U test after Shapiro-Wilk normality test.

SPSS software (version 21.0, IBM Corp., Armonk, NY, USA) and MedCalc software (version 17.0, MedCalc Software bvba, Mariakerke, Belgium) were used for statistical analyses. A *P*-value < 0.05 was considered significant.

## Results

A total of 709 patients were reviewed for eligibility and 635 patients were included in the regression analysis after excluding 74 patients who took analgesic medication due to acute and chronic pain (5 cases), participated in another clinical trial (60 cases), and in whom NRS record was missing (9 cases).

**Fig 1:**
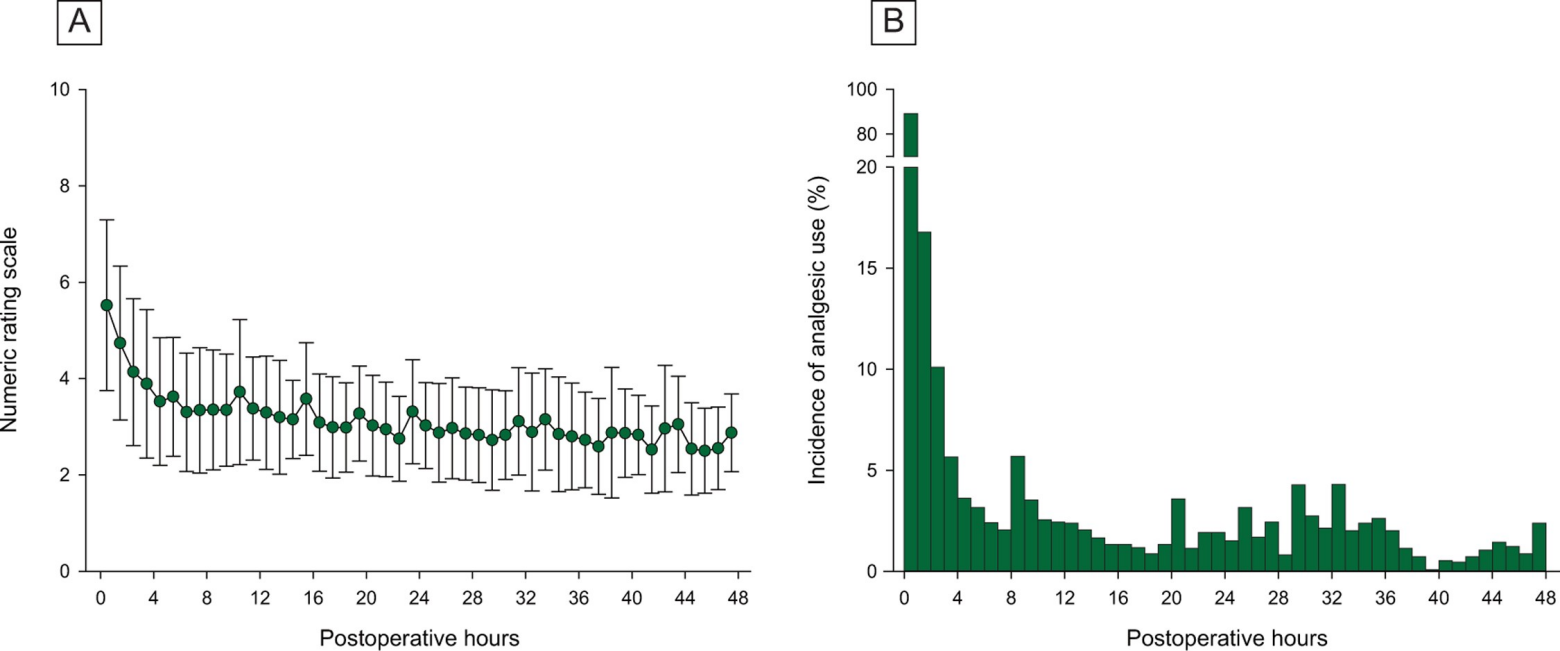
Changes in pain scores and analgesic consumption for 48 hours after robotic thyroidectomy. A: Postoperative pain is quantified by 11-point (0–11) numeric rating scale. Data are expressed as mean ± SD (symbol and error bar). B: The number of patients requiring analgesic for each hour is divided by the total number of patients. Data are expressed as incidence (%).

Patient characteristics are described in Table 1. The cumulative doses of propofol and remifentanil were 1840 mg and 2100 mcg, respectively, during 3.8 hours’ anesthesia. The frequency and cumulative dose of analgesic per patient were 2 times and 1.0 MED10, respectively, during 48 hours after surgery. Fig 1 shows the changes of NRS and MED10 every hour during 48 postoperative hours in all patients. Within 1 hour after surgery, analgesic was administered in 89.1% (566/635) and incidence of treatment-requiring pain was 67.5% (429/635) with a median NRS of 5.5. At 48 hours, analgesic was still administered in 2.4% of patients (15/635) and treatment-requiring pain persisted in 12.8% (81/635) with median NRS of 1.5.

**Table 1:** Basic characteristics (n = 635).

Variable	Value
Age (years)	40 (33–48)
Gender (M/F)	74/561
Weight (kg)	58 (53–66)
Height (cm)	161 (158–166)
Surgical duration (hours)	3.8 (3.4–4.3)
Propofol cumulative dose (mg)	1840 (1505–2200)
Remifentanil cumulative dose (mcg)	2100 (1650–2700)
Postoperative pain score	
Postoperative 1 hour	5.5 (4.3–6.0)
Postoperative 48 hours	1.5 (0–3.0)
Analgesic consumption	
Frequency	2 (1–3)
Cumulative dose (MED10)	1.0 (0.5–2.5)

Data are median (interquartile range) or number (%). Pain score was rated using 11-point (0–10) numeric rating scale. The amount of analgesic consumption was calculated as MED10 during 48 postoperative hours. MED10 = morphine 10 mg equivalent dose.

Multivariable Cox proportional hazards regression analyses identified analgesic consumption and intraoperative use of remifentanil > 0.2 mcg/kg/min as negative and positive predictors, respectively. However, Spearman correlation tests showed that the effects of analgesic consumption and remifentanil category on the presence of pain were time-dependent (*P* < 0.049 and *P* < 0.001, respectively). Therefore, time dependent Cox proportional hazards regression analysis was performed, and the test identified that intraoperative use of remifentanil > 0.2 mcg/kg/min, analgesic consumption, and the interaction of analgesic consumption and time were significant predictors of treatment-requiring pain during 48 hours after surgery (Table 2).

**Table 2:** Predictors of postoperative treatment-requiring pain in robotic thyroid surgery patients anesthetized with total intravenous anesthesia.

Predictor	Adjusted hazard ratio (95% confidence interval)	*P*-value
Remifentanil infusion rate > 0.2 mcg/kg/min	1.296 (1.014–1.656)	0.039
Analgesic consumption (per MED10)	0.782 (0.719–0.085)	< 0.001
Analgesic consumption by time (MED10 hour)	1.005 (1.001–1.009)	0.024

Treatment-requiring pain was defined when 11-point numeric rating scale (0–11 point) of the pain is greater than 4. Analgesic consumption was calculated as MED10 during 48 postoperative hours. MED10 = morphine 10 mg equivalent dose.

Finally, after adjusting for the time dependent analgesic consumption, intraoperative use of remifentanil > 0.2 mcg/kg/min was a positive predictor of treatment-requiring pain with a hazard ratio of 1.296 (95% confidence interval, 1.014–1.656, *P* = 0.039) during 48 hours after surgery.

**Fig 2:**
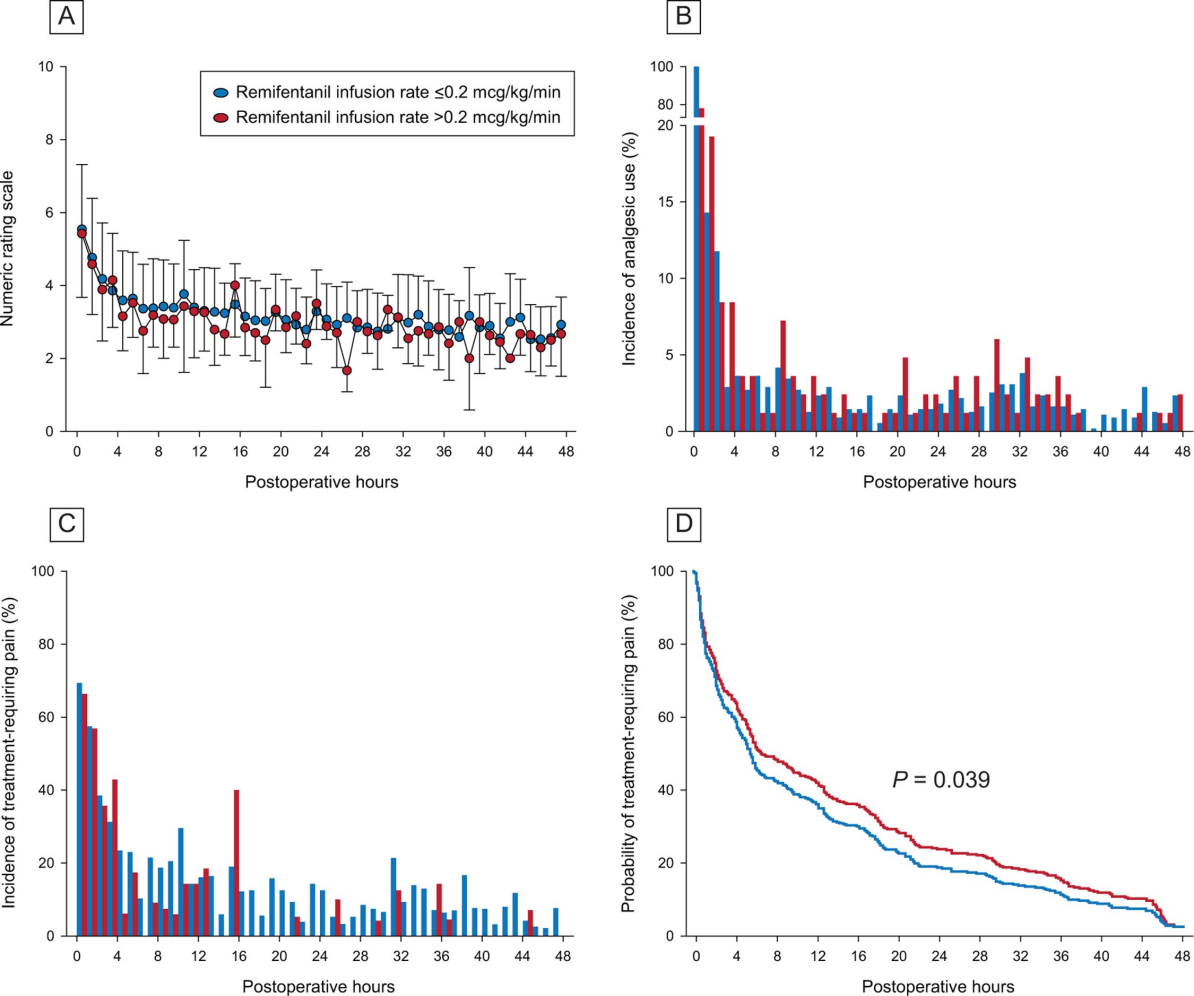
Changes in pain scores, analgesic consumption, and incidence of treatment-requiring pain in two remifentanil groups for 48 hours after robotic thyroidectomy. Postoperative pain was quantified by 11-point (0–11) numeric rating scale. Treatment-requiring pain was defined when numeric rating scale of the pain is greater than 4. A, B and C: Two remifentanil categories are not easily distinguishable in terms of pain scores, analgesic use and treatment-requiring pain incidence. D: Time dependent Cox proportional hazards regression analysis identified that the risk of treatment-requiring pain was 1.3 times higher in the high-dose remifentanil group than in the low-dose group after adjusting for analgesic consumption and its interaction with time.

*Post-hoc* comparison showed that weight-adjusted propofol dose and propofol infusion rate were significantly higher in patients with remifentanil > 0.2 mcg/kg/min than those with remifentanil ≤ 0.2 mcg/kg/min (Table 3). However, two groups were not different regarding postoperative NRS and analgesic consumption. Fig 2A and 2B show the changes of NRS and MED10, respectively, during 48 postoperative hours according to the remifentanil ≤ 0.2 mcg/kg/min and remifentanil > 0.2 mcg/kg/min groups. Fig 2C and 2D show the actual incidence and predicted probability of treatment-requiring pain, respectively, in the two groups. The probability of treatment-requiring pain was 1.3 times higher in the remifentanil > 0.2 mcg/kg/min group than that in the remifentanil ≤ 0.2 mcg/kg/min group throughout the postoperative 48 hours when adjusted by postoperative analgesic consumption.

**Table 3:** Characteristics of remifentanil infusion rate groups.

Variable	Remifentanil infusion rate ≤ 0.2 mcg/kg/min (n = 552)	Remifentanil infusion rate > 0.2 mcg/kg/min (n = 83)	*P*-value
Age (years)	41 (34–49)	35 (27–44)	< 0.001
Gender (M/F)	69/483	5/78	0.099
Weight (kg)	58 (53–67)	54 (50–60)	< 0.001
Height (cm)	161 (158–166)	161 (157–163)	0.061
Surgical duration (hours)	3.8 (3.4–4.3)	3.9 (3.6–4.4)	0.313
Propofol			
Cumulative dose (mg)	1840 (1500–2200)	2000 (1600–2160)	0.612
Weight-adjusted dose (mg/kg)	30.7 (26.6–36.1)	35.3 (29.9–39.3)	< 0.001
Infusion rate (mg/kg/min)	0.13 (0.12–0.15)	0.15 (0.13–0.16)	< 0.001
Remifentanil			
Cumulative dose (mcg)	2000 (100–2500)	3000 (2500–3245)	< 0.001
Weight-adjusted dose (mcg/kg)	34.3 (27.3–40.8)	52.9 (47.3–60.1)	< 0.001
Infusion rate (mcg/kg/min)	0.15 (0.13–0.17)	0.22 (0.21–0.24)	< 0.001
Postoperative pain score			
Postoperative 1 hour	5.5 (4.5–6.0)	5.0 (4.0–6.0)	0.643
Postoperative 48 hours	1.5 (0–3.0)	2.0 (0–2.5)	0.854
Average	3.9 (3.3–4.7)	3.8 (3.2–4.3)	0.174
Analgesic consumption			
Frequency	2 (1–3)	2 (1–3)	0.430
Cumulative dose (MED10)	1.0 (0.5–2.5)	1.5 (0.9–2.7)	0.353

Data are median (interquartile range) or number (%). Pain score was rated using 11-point (0–10) numeric rating scale. The amount of analgesic consumption was calculated as MED10 during 48 postoperative hours. MED10 = morphine 10 mg equivalent dose.

## Discussion

In the current study, we identified that both intraoperative use of remifentanil and postoperative use of analgesics were closely related with postoperative pain scores after robotic thyroid surgery. After adjusting for the analgesic use, the risk of pain within 48 postoperative hours was 1.3 times greater in the patients who used more remifentanil (> 0.2 mcg/kg/min) than less remifentanil intraoperatively.

Specific reasons why adult robotic thyroid surgery patients were selected for our retrospective study need to be addressed in advance. First, due to the increased use of intraoperative monitoring of recurrent laryngeal nerve, TIVA, which is known to minimally affect nerve monitoring [16], has become the routine protocol of anesthesia for robotic thyroid surgery. In addition, high dose opioids are frequently used to ensure immobility when muscle relaxants are omitted for nerve monitoring. The relatively longer operation time also contributes to use of large dose remifentanil. Therefore, this subset of patients is likely to be associated with excessive use of remifentanil in TIVA and is suitable to assess the end point of our study. Second, the pain after robotic thyroid surgery is localized to the anterior chest and not aggravated by movement [17]. The pain tends to be moderate to minor and rapidly disappears with minimal analgesics [12]. We hypothesized that abnormally increased pain after thyroid surgery could be rather easily detected. Finally, since most of the patients are in their 30s and 40s, exclusion due to chronic pain and analgesic medication could be minimized.

The remifentanil-associated AOT/OIH is poorly understood and controversial in TIVA especially in TCI mode as shown in Table 4 [6–10]. We suppose that the major reason for the argument is the difference in the way the threshold is expressed. Although the thresholds of remifentanil infusion rates reported in previous studies are directly comparable, the Ce thresholds reported in the TCI studies are not. According to the pharmacokinetic model of Minto [18], the Ce of remifentanil is strongly influenced by age as well as body weight. For example, the TCI group in Richebé’s study had an average age of 66 years and weighed 70 kg, and the Ce and an average infusion rate calculated from the total amount of remifentanil use and infusion duration was 7 ng/mL and 0.19 mcg/kg/min, respectively [8]. In their study, despite the high Ce, the infusion rate did not exceed the threshold of our study, which is presumed to be the cause of low incidence of pain. In two studies reporting no significant difference in pain incidence, the values of Ce 1.0 ng/mL and Ce 4.0 ng/mL set for the control and experimental groups, respectively, did not exceed the threshold infusion rate of 0.2 mcg/kg/min [6, 9]. Two other studies reporting that remifentanil-related pain occurred even when remifentanil was used below the threshold additionally used fentanyl as well as remifentanil, but they did not provide the exact amount of fentanyl [7, 10]. Based on our findings, a similar conclusion to the reports of inhalation anesthetics can be drawn that increased pain may occur when the remifentanil infusion rate, not Ce, exceeded 0.2 mcg/kg/min.

**Table 4:** Studies on remifentanil-associated pain after total intravenous anesthesia with propofol and remifentanil.

Author (year)	Surgery	Group	N	Mode of infusion	Remifentanil target concentration (ng/mL)	Infusion rate (mcg/kg/min)	Outcome
Rauf (2005) [10]	Off-pump coronary artery surgery	control	10	Placebo	NA	NA	Greater analgesic consumption in the test group
		test	10	CI	NA	0.10	
Shin (2010) [9]	Breast cancer surgery	control	50	TCI	1	0.06*	No difference
		test	46	TCI	4	0.15*	
Richebé (2011) [8]	Elective cardiac surgery	control	19	CI	NA	0.30	More hyperalgesia in the control group
		test	19	TCI	7*	0.19*†	
Jo (2011) [7]	Total abdominal hysterectomy	control	20	Placebo	NA	NA	Greater analgesic consumption in the test group
		test	20	TCI	3–4	0.11	
Koo (2016) [6]	Pancreatico-duodenectomy	control	27	TCI	1	0.04	No difference between groups
		test	26	TCI	4	0.14	

All studies are randomized controlled trials.

* Infusion rate in TCI mode was calculated using total consumption of remifentanil, anesthesia duration and patient’s weight presented in the report.

† The authors described that the target concentration of 7 ng/mL was equivalent to the infusion rate of 0.3 mcg/kg/min, however the average infusion rate calculated from the presented data is 0.19 mcg/kg/min.

Abbreviations: CI = continuous infusion; NA = not applicable; TCI = target-controlled infusion

Compared with inhalation anesthesia, reducing remifentanil infusion rate below the threshold during TIVA may require some additional considerations. Increasing propofol may cause systemic hypotension through dose-dependent arterial relaxation and myocardial suppression [19]. After prolonged use of propofol, recovery from anesthesia can be delayed because of increased context-sensitive decrement time [20]. Excessive propofol and consequently low BIS may increase postoperative morbidity and mortality [21]. Finally, the current anesthetic strategy of low propofol-high remifentanil during TIVA may not achieve the protective effect of propofol mentioned in previous reports [22]. In the current study, we failed to confirm the protective effect of propofol through regression analysis. There was no substantial difference in the amount of propofol used between the two remifentanil groups and the amount of propofol itself was small. We claim that it is better to use other non-opioid adjuncts such as N-methyl-D-aspartate (NMDA) receptor antagonists and non-steroidal anti-inflammatory drugs than to increase propofol usage in order to reduce the risk of remifentanil-associated pain in TIVA [2].

Our retrospective study is better than previous studies in the following aspects. First, previous studies compared the postoperative pain scores and analgesic consumption separately at vague time points [2]. However, given the mechanism of remifentanil-associated pain, the patient must be continuously exposed to the increased risk of pain from the moment the remifentanil dose exceeded the threshold. We assumed that pain scores and the effect of analgesics are interdependent and time-dependent. The changes in scores and incidence of pain over time illustrated in Fig 2A and 2C can be the result of time-dependent use of analgesics shown in Fig 2B. In our study, time dependent Cox regression analysis successfully solved the issue of interdependence among pain incidence, analgesic use and time. Second, most of prospective studies included less than 100 patients. Negative reports on the incidence of remifentanil-associated pain may be due to the small sample size in part. Our *post-hoc* power analysis suggested that more than 4000 cases per group are required to reveal significance difference in analgesic consumption between the two groups using T-test. The benefit of this study is that we have chosen the appropriate statistical method as well as large sample size. In our study using more than 600 subjects, moderate difference such as a hazard ratio of 1.3 could be revealed with sufficient power using regression analysis.

Our study has some limitations. First, a recent review of remifentanil-related AOT/IOH suggests that the weight-adjusted dose of more than 50 mcg/kg is the most obvious cutoff [3]. However, weight-adjusted remifentanil dose > 50 mcg/kg was not a threshold for increased pain in our study. We additionally performed an agreement analysis and identified that the Kappa statistic between the cases of remifentanil infusion rate > 0.2 mcg/kg/min and weight-adjusted remifentanil dose > 50 mcg/kg was only moderate (0.526, 95% confidence interval, 0.430–0.621). In fact, the weight-adjusted dose does not distinguish between excessive dose over a short period and moderate dose over a longer period. Standardization of the cumulative dose to the duration of administration should be warranted in future studies. Second, infusion rate of 0.2 mcg/kg/min was a significant criterion, but 0.25 mcg/kg/min was not, indicating that the cutoff might have been misconfigured. This is probably due to the fact that the number of subjects in the latter was only six, which is too small to have enough power. In order to observe the linear increase in remifentanil-associated AOT/OIH risk with increasing remifentanil usage, further studies that include cases requiring higher doses of remifentanil during surgery may be required.

In conclusion, our retrospective study identified that infusion of remifentanil > 0.2 mcg/kg/min during TIVA increases the probability of treatment-requiring pain for 48 hours after robotic thyroid surgery, when adjusting for analgesic consumption and its interaction with time. We suggest that care should be taken to avoid excessive use of remifentanil during TIVA because no evidence regarding the protective effect of propofol was found in our study. However, prospective trials are required to support our results and determine whether decreasing intraoperative remifentanil consumption below the threshold can reduce postoperative pain.

## Supporting information

S1 File Raw data for this study including patient characteristic, amount of anesthetic agent consumption, and reported NRS after surgery. (XLSX)
